# Molecular Epidemiology of Methicillin-Resistant *Staphylococcus aureus* Isolated from Australian Veterinarians

**DOI:** 10.1371/journal.pone.0146034

**Published:** 2016-01-06

**Authors:** Mitchell D. Groves, Bethany Crouch, Geoffrey W. Coombs, David Jordan, Stanley Pang, Mary D. Barton, Phil Giffard, Sam Abraham, Darren J. Trott

**Affiliations:** 1 School of Veterinary and Life Sciences, Murdoch University, Murdoch, Australia; 2 School of Pharmacy and Medical Sciences, University of South Australia, Adelaide, South Australia, Australia; 3 Department of Microbiology, Path West Laboratory Medicine, WA, Fiona Stanley Hospital, Murdoch, Western Australia, Australia; 4 New South Wales Department of Primary Industries, Wollongbar, New South Wales, Australia; 5 Menzies School of Health Research, Casuarina, Northern Territory, Australia; 6 School of Animal and Veterinary Sciences, The University of Adelaide, Roseworthy, South Australia, Australia; University Hospital Münster, GERMANY

## Abstract

This work investigated the molecular epidemiology and antimicrobial resistance of methicillin-resistant *Staphylococcus aureus* (MRSA) isolated from veterinarians in Australia in 2009. The collection (*n* = 44) was subjected to extensive molecular typing (MLST, *spa*, SCC*mec*, *dru*, PFGE, virulence and antimicrobial resistance genotyping) and antimicrobial resistance phenotyping by disk diffusion. MRSA was isolated from Australian veterinarians representing various occupational emphases. The isolate collection was dominated by MRSA strains belonging to clonal complex (CC) 8 and multilocus sequence type (ST) 22. CC8 MRSA (ST8-IV [2B], *spa* t064; and ST612-IV [2B], *spa* variable,) were strongly associated with equine practice veterinarians (OR = 17.5, 95% CI = 3.3–92.5, *P* < 0.001) and were often resistant to gentamicin and rifampicin. ST22-IV [2B], *spa* variable, were strongly associated with companion animal practice veterinarians (OR = 52.5, 95% CI = 5.2–532.7, *P* < 0.001) and were resistant to ciprofloxacin. A single pig practice veterinarian carried ST398-V [5C2], *spa* t1451. Equine practice and companion animal practice veterinarians frequently carried multiresistant-CC8 and ST22 MRSA, respectively, whereas only a single swine specialist carried MRSA ST398. The presence of these strains in veterinarians may be associated with specific antimicrobial administration practices in each animal species.

## Introduction

Since the turn of the millennium our understanding of the ecological niches and genetic diversity of methicillin-resistant *Staphylococcus aureus* (MRSA) has changed markedly. Several distinct lineages of MRSA are now known to inhabit and cause clinical infections in animals including horses, companion animals (i.e. dogs and cats) and intensively managed livestock [1,2]. These so-called “animal-adapted” MRSA are a concern for public health as, under certain circumstances, they can colonise and/or cause infections in humans with intimate exposure to animals [1,2].

Several studies have shown human healthcare workers (HCWs) working with MRSA-colonised patients and veterinarians with routine occupational exposure to animals often have a higher prevalence of MRSA nasal colonisation in comparison to the general public [3,4]. For instance, the prevalence of ST398 MRSA colonisation amongst livestock veterinarians in Europe is estimated to be greater than 40%, [5,6] and the prevalence of CC8 MRSA colonisation amongst equine veterinary personnel in North America ranges from 9.7% to 18% [7–9]. Moreover, ST22 MRSA has been recovered from 17.9% of companion animal veterinarians in the United Kingdom [10]. In a recent Australian study, the prevalence of MRSA nasal colonisation was found to be extremely high among specialist equine veterinarians (21.4%) and well above the average for those veterinarians practicing companion animal medicine only (4.9%) [11]. In human medicine in a review of 127 outbreak studies, 4.6% of 33,318 HCWs working with MRSA-positive patients were colonised with MRSA [3]. In a recent nasal MRSA colonisation prevalence study on HCWs working in a Western Australian acute care hospital only 3.4% of 1,542 HCWs screened were MRSA colonised. However, 10.7% of HCWs working in high risk MRSA wards were colonised [12].

In human medicine the prevalence of HCW colonisation has been shown to be dependent upon the circulating MRSA strain, with different strains varying in their ability to colonise HCWs [13]. While previous studies have identified and characterised MRSA strains from horses (CC8), dogs (ST22) and pigs (ST398) in Australia [14–16]. In our previous study evaluating the prevalence of MRSA carriage among Australian veterinarians, we identified those veterinarians with horses as a major area of work emphasis had a prevalence of 11.8% and those whose only major emphasis was horses had a prevalence of 21.4%. Veterinarians with dogs and cats as a major activity had a 4.9% prevalence. Whilst prevalence rates for other major activities such as pigs (8.3%), dairy (4.6%), beef cattle (8.1%), avian (10%) and wildlife (3.23%) were also increased, however these were estimated from smaller numbers of respondents [11].

In the current study we sought to define the molecular epidemiology of MRSA isolated from Australian veterinarians from our previous study to better understand why some veterinarians have a high prevalence of MRSA [11]. This study also aims to place these strains into epidemiological context with strains from other sources (e.g. animals, community-associated and healthcare-associated strains), both in Australia and abroad.

## Materials and Methods

### Bacterial isolates

The study characterised 44 MRSA isolates recovered from the anterior nares of 771 healthy veterinarians sampled during a series of national veterinary conferences in Australia in 2009 [11]. As previously described, nasal swabs were obtained from veterinarians attending the Australian Veterinary Association Annual Conference in Darwin (17–22 May 2009), the Australian Pig Veterinarians Conference in Melbourne (22–23 June 2009), the Australian College of Veterinary Scientists Conference on the Gold Coast (2–4 July 2009) and the Bain Fallon Equine Conference at the Sunshine Coast (20–24 July 2009) [11]. The recruitment of human subjects into this study was approved by the Human Research Ethics Committee of the Northern Territory Department of Health & Families and Menzies School of Health Research (HREC 09/41). Those who volunteered were provided with background reading on MRSA and recruitment was formalised by obtaining signed consent. Each subject was assigned a coded, unique number and adhesive labels displaying this number were each affixed to the information sheet (retained by each subject), consent form, sterile cotton tipped swab and a receptacle for the swab containing sterile enrichment broth. For the duration of the study, researchers were blinded to the identity of respondents, and respondents who tested positive for MRSA and indicated that they wanted to be informed of their culture results were confidentially notified. Whilst participants completed an online questionnaire as part of the survey, as per the conditions of the human ethics approval (HREC 09/41), their identity and location of practice remained anonymous.

The three major veterinarian occupations that tested positive for MRSA were: equine practice (EP, *n* = 18), companion animal practice (CAP, *n* = 14), and mixed animal practice with both companion animals and equines (MPE, *n* = 6). Six MRSA were isolated from mixed animal practice with companion animals and ruminants but no equines (MP, *n* = 2), pig practice (PP, *n* = 1), ruminant herd consulting practice (HC, *n* = 1), industry veterinarian (pharmaceutical representative) with no clinical practice (IND, *n* = 1), and a veterinary science student with undefined exposure (STU, *n* = 1).

### *nuc* and *mecA* characterization

*Staphylcoccus aureus* species and methicillin resistance were confirmed by the detection of *nuc* (thermostable extracellular nuclease) and *mecA* (methicillin resistance) genes respectively using multiplex PCR. [17]

### Antimicrobial susceptibility testing

Antimicrobial susceptibility testing was performed by disk diffusion according to the Clinical and Laboratory Standards Institute (CLSI) recommendations [18]. A panel of ten antimicrobial agents was tested: cefoxitin (30μg), chloramphenicol (30μg), ciprofloxacin (5μg), clindamycin (2μg), erythromycin (15μg), fusidic acid (10μg), gentamicin (10μg), mupirocin (5μg) rifampicin (5μg), tetracycline (30 μg), and trimethoprim (5 μg). CLSI interpretive criteria were used for all antimicrobials except fusidic acid and mupirocin. The European Committee on Antimicrobial Susceptibility Testing (EUCAST) break points were used for fusidic acid and mupirocin.[19] Isolates displaying resistance to three or more antimicrobial classes were considered to be multidrug-resistant (MDR).

### Pulsed-field gel electrophoresis (PFGE)

Electrophoresis of chromosomal DNA was performed on all isolates as previously described, using a contour-clamped homogeneous electric field (CHED) DR III system [20]. Chromosomal patterns were examined visually, scanned with a Quantity One (Bio-Rad) and digitally analysed using FPQuest (Bio-Rad). *S*. *aureus* strain NCTC 8325 was used as a reference strain.

### DNA microarray

All isolates were analysed using a *S*. *aureus* DNA microarray. Arrays and reagents were obtained from Alere Technologies, Jena Germany. The principle of the assay, related procedures, and a list of targets have been described previously [21]. The microarray was used to detect the presence of virulence and antimicrobial resistance genes. Probes for *mecA*, *ugpQ*, *xylR*, and two probes for *mecR* were used for SCC*mec* typing. The last two probes allowed detection and discrimination for untruncated *mecR* and Δ*mecR*, respectively. Probes for the recombinase genes *ccrA1*, *ccrB1*, *ccrA2*, *ccrB2*, *ccrA3*, *ccrB3*, *ccrA4*, *ccrB4* and *ccrC1*; the fusidic acid resistance marker Q6GD50; and the J region proteins, *dcs*, *plsSCC* and the *kdp*-operon also were included. Ambiguous array results were considered negative.

### *spa* and *dru* typing

Sequence analysis of the protein A gene variable region (*spa*) and the *mec*-associated *dru* region was performed on all isolates as previously described [22,23]. Chromosomal DNA was prepared using a DNeasy (Qiagen) tissue kit.

### Multilocus sequence typing (MLST)

The MLST for each isolate with a unique PFGE pulsotype or s*pa* type was determined by Illumina Miseq. Genomic DNA libraries were prepared using the Nextera XT kit (Illumina) and sequenced on the 250-bp pair-ended chemistry. DNA sequences collated at http://saureus.mlst.net/ belonging to 3,044 ST types were downloaded in FASTA format and used as the database in the SRST2 pipeline to match the corresponding MLST profiles to the Illumina sequence data for each isolate [24].

Isolates sharing six of seven MLST loci were deemed to belong to the same clonal complex (CC). Double locus variants were included in the same CC only when the linking single locus variant was also present in the MLST database (http://www.mlst.net/).

### Statistical analyses

The strength of association between major occupational emphasis and MRSA clonal complex recovered from veterinarians was assessed by calculating the odds ratio (OR) with 95% confidence intervals (95% CI). An OR greater than unity represents a positive association, while OR less than unity represents a negative association. The magnitude of departure of OR from unity represents the strength of association. P-values (*P*) for assessing the null hypothesis of nil-association between virulence and isolate origin (OR = 1) were calculated using Fisher’s exact test and differences were considered significant when *P* ≤ 0.05.

## Results

### MLST, PFGE, *spa*, SCC*mec* and *dru* types

The isolate collection comprised eight multilocus sequence types (STs) and seven clonal complexes (CCs) (S1 File). The clonal lineages included CC1, CC5, CC8, CC22, CC59, CC88, and CC398 (Table 1). Seventeen *spa* types were detected and ten different SCC*mec* and *dru* combinations were observed, with SCC*mec* IV [2B]-dt10a being most dominant among the collection (27/44 isolates, 61.4%). The predominant clonal lineages were CC8 (24/44, 54.5%) and CC22 (12/44, 27.3%). CC8 was recovered most frequently from equine only veterinarians (15/18) and included two STs; ST8 (20/24) and ST612 (4/24). All CC8 isolates harboured SCC*mec* IV [2B] and had closely related s*pa* and *dru* types, with *spa* t064 *dru* dt10a being most dominant, and by PFGE had a WA20 pulsotype. CC22 was most frequently isolated from companion animal veterinarians (10/14, 71.4%) and consisted of one ST, ST22, which harboured SCC*mec* IV [2B]. The isolates were predominantly *spa* type t032, *dru* type dt10a and had an EMRSA-15 PFGE pulsotype. A single isolate from a pig practice veterinarian was identified as ST398-V [5C2] (*spa* t1451, *dru* dt11a).

**Table 1:** Molecular characteristics of methicillin-resistant *Staphylococcus aureus* (*n* = 44) isolated from veterinarians in Australia.

Isolate	PFGE	*spa*	ST (CC)	SCC*mec*	*dru*	Resistance phenotype
**Companion Animal Practice (14)**						
34	EMRSA15	t032	22 (22)	IV [2B]	dt10a	CIP
253	EMRSA15	t032	22 (22)	IV [2B]	dt10a	CIP
257	EMRSA15	t032	22 (22)	IV [2B]	dt10a	ERY, CIP
330	EMRSA15	t032	22 (22)	IV [2B]	dt10a	CIP
844	EMRSA15	t032	22 (22)	IV [2B]	dt10a	CIP
428	EMRSA15	t1625	22 (22)	IV [2B]	dt10bw	CIP
610	EMRSA15	t3547	22 (22)	IV [2B]	dt10a	CIP
622	EMRSA15	t9448	22 (22)	IV [2B]	dt10a	CIP
38	EMRSA15	t10924	22 (22)	IV [2B]	dt10a	CIP
606	EMRSA15	t10924	22 (22)	IV [2B]	dt10a	CIP
616	USA100	t242	5 (5)	II [2A]	NIL	ERY, CLI, CIP
580	WA20	t064	8 (8)	IV [2B]	dt10a	GEN, TET, TRM
673	WA20	t1171	8 (8)	IV [2B]	dt9j	GEN, ERY, TET, RIF
612	WA20	t064	8 (8)	V [5C2]	dt9aw	GEN, TET, TRM
**Equine Practice (18)**						
809	EMRSA15	t1977	22 (22)	IV [2B]	dt10a	CIP
728	WA2	t186	78 (88)	IV [2B]	dt10a	ERY
815	WA2	t186	78 (88)	IV [2B]	dt9ah	ERY
713	WA20	t064	8 (8)	IV [2B]	dt10a	GEN, TET, TRM, RIF
699	WA20	t064	8 (8)	IV [2B]	dt10a	GEN, TET, TRM, RIF
718	WA20	t064	8 (8)	IV [2B]	dt10g	GEN, ERY, TET, TRM, RIF
727	WA20	t064	8 (8)	IV [2B]	dt10a	GEN, TET, TRM
741	WA20	t064	8 (8)	IV [2B]	dt9j	GEN, ERY, TET, TRM, RIF
761	WA20	t064	8 (8)	IV [2B]	dt10a	GEN, TET, TRM
783	WA20	t064	8 (8)	IV [2B]	dt10a	GEN, TET, RIF
787	WA20	t064	8 (8)	IV [2B]	dt10a	GEN, ERY, TET, TRM, RIF
823	WA20	t064	8 (8)	IV [2B]	dt10a	GEN, ERY, TET, TRM, RIF
58	WA20	t2658	8 (8)	IV [2B]	dt10a	GEN, TET, TRM, RIF
734	WA20	t064	8 (8)	IV [2B]	dt10a	GEN, ERY, TET, TRM, RIF,
742	WA20	t064	8 (8)	IV [2B]	dt7d	GEN, ERY, TET, TRM, RIF
822	WA20	t451	612 (8)	IV [2B]	dt7d	GEN, TET, TRM, RIF
723	WA20	t723	612 (8)	IV [2B]	dt7d	GEN, ERY, TET, TRM, RIF
778	WA20	t723	612 (8)	IV [2B]	dt7d	GEN, ERY, TET, TRM, RIF
**Herd Consulting Practice (1)**						
786	WA2	t186	78 (88)	IV [2B]	dt9ah	ERY
**Industry Veterinarian—Pharmaceutical Representative (1)**						
55	WA20	t064	8 (8)	IV [2B]	dt10a	ERY, TET, TRM
**Mixed Animal Practice (2)**						
389	WA20	t064	8 (8)	IV [2B]	dt10a	GEN, ERY, TET, TRM, RIF
570	WA20	t064	612 (8)	IV [2B]	dt7d	GEN,TET, TRM, RIF
**Mixed Animal Practice with both Companion Animals and Equines (6)**						
100	EMRSA15	t032	22 (22)	IV [2B]	dt10a	CIP
773	WA15	t976	59 (59)	IV [2B]&5	dt10a	ERY
103	WA2	t690	78 (88)	IV [2B]	dt10a	
747	WA20	t064	8 (8)	IV [2B]	dt10a	GEN, ERY, TRM
413	WA20	t064	8 (8)	IV [2B]	dt7d	GEN, ERY, TET, TRM, RIF
716	WA20	t064	8 (8)	IV [2B]	dt7d	GEN, TET, TRM, RIF
**Pig Practice (1)**						
516	ST398	t1451	398 (398)	V [5C2]	dt11a	ERY, TET, TRM, CIP, CLI
**Veterinary Science Student with Undefined Exposure (1)**						
698	WA1	t1853	1 (1)	IV [2B]	dt10a	

PFGE: pulsed-field gel electrophoresis pulsotype; *spa*: staphylococcal protein A type; ST: multilocus sequence type; CC: clonal complex; SCC*mec*, staphylococcal chromosome cassette *mec* type; *dru*: direct repeat unit type; CIP: ciprofloxacin; CLI: clindamycin; ERY: erythromycin; GEN: gentamicin; RIF: rifampicin; TET: tetracycline; TRM: trimethoprim.

Mixed practice veterinarians including those with exposure to both companion animals and equines were found to carry ST22-IV [2B] (1/6), ST8-IV [2B] (3/6), ST59-IV [2B] (1/6) and ST78-IV [2B] (1/6). CC8 (ST8-IV [2B] and ST612-IV [2B]) also was carried by mixed practice veterinarians with companion and bovine but no equine exposure and an industry veterinarian who was not exposed to clinical veterinary practice. A veterinary student with undefined exposure carried ST1-IV [2B] and a herd consulting veterinarian carried ST78-IV [2B].

### Antimicrobial resistance phenotypes and genotypes

More than two-thirds of isolates (32/44, 72.7%) were MDR with resistance influenced by the clone type; ST22-IV [2B] (PFGE EMRSA-15) was typically ciprofloxacin-resistant and ST8-IV [2B] (PFGE WA20) was typically gentamicin, tetracycline, erythromycin and rifampicin-resistant (S1 File). Resistance to erythromycin (20 isolates) was conferred by *erm(A)* (4 isolates) *erm(C)* (15), and *msr(A)*[macrolide efflux pump]*/ mph(C)*[macrolide phosphotransferase] (1) (Tables 1 and 2). Tetracycline resistance (24 isolates) was conferred by *tet(K)* (2), *tet(M)* (23). One tetracycline-resistant isolate harboured *tetK* and *tetM* genes. Twenty one of the 23 trimethoprim-resistant isolates harboured *dfr* (dihydrofolate reductase mediating trimethoprim resistance). Isolates from equine-specialist veterinarians were frequently resistant to gentamicin (15/18) and rifampicin (10/18). Gentamicin resistance was conferred by *aacA-aphD* (aminoglycosideadenyl-/phosphotransferase) (15). Three gentamicin-resistant isolates also harboured *aadD (aminoglycoside adenyltransferase*). The mechanism conferring rifampicin resistance was not confirmed. Only four of the isolates from companion animal veterinarians were MDR, however, almost all isolates were ciprofloxacin-resistant (11/14). Whole genome sequencing showed ciprofloxacin resistance was due to two point mutations generating from amino acid substitutions Ser80Phe in topoisomerase IV (Grla) and Ser84Leu in gyrase A (GyrA). None of the isolates carried *vanA*, *vanB* or *vanZ* glycopeptide-resistant genes. Furthermore, no isolates tested positive for the resistance determinants *lnu*(A) (lincosamides), *mef*(A) (macrolides), *vat*(A) (virginiamycin), *vga*(A) (streptogramin A, lincosamide, pleuromutilin), *vgb*(A) (virginiamycin-B/pristinamycin), *mupA* (mupirocin), *cat* (chloramphenicol), or *fexA* (phenicols).

**Table 2:** Antimicrobial resistance and virulence genotypes of methicillin-resistant *Staphylococcus aureus* strains (*n* = 44) isolated from veterinarians in Australia.

Isolate	Origin	Resistance genotype	Virulence genotype
**Clonal Complex 1**			
698	STU	*blaZ-I-R*, *sdrM*	*seh*, *sak*, *chp*, *scn*
**Clonal Complex 5**			
616	CAP	*blaZ-I-R*, *erm(A)*, *aadD*, *sdrM*, *fosB*	*sed*, *seg*, *sei*, *sej*, *sem*, *sen*, *seo*, *seq*, *ser*, *seu*
**Clonal Complex 8**			
580	CAP	*blaZ-I-R*, *aacA-aphD*, *dfrA*, *tet(M)*, *sdrM*, *fosB*	*sea*, *seb*, *sek*, *seq*, *sak*, *scn*
673	CAP	*blaZ-I-R*, *erm(C)*, *aacA-aphD*, *tet(M)*, *sdrM*, *fosB*	*sea*, *seb*, *sek*, *seq*, *sak*, *scn*
713	EP	*blaZ-I-R*, *aacA-aphD*, *dfrA*, *tet(M)*, *sdrM*, *fosB*	*seb*, *sek*, *seq*
699	EP	*blaZ-I-R*, *aacA-aphD*, *aadD*, *dfrA*, *tet(M)*, *sdrM*, *fosB*	*sea*, *seb*, *sek*, *seq*, *sak*, *scn*
718	EP	*blaZ-I-R*, *erm(C)*, *aacA-aphD*, *aadD*, *dfrA*, *tet(M)*, *sdrM*, *fosB*	*sea*, *seb*, *sek*, *seq*, *sak*, *scn*
727	EP	*blaZ-I-R*, *aacA-aphD*, *dfrA*, *tet(M)*, *sdrM*, *fosB*	*sea*, *seb*, *sek*, *seq*, *sak*, *scn*
741	EP	*blaZ-I-R*, *erm(C)*, *aacA-aphD*, *dfrA*, *tet(M)*, *sdrM*, *fosB*	*sea*, *seb*, *sek*, *seq*, *sak*, *scn*
761	EP	*blaZ-I-R*, *aacA-aphD*, *dfrA*, *tet(M)*, *sdrM*, *fosB*	*sea*, *seb*, *sek*, *seq*, *sak*, *scn*
783	EP	*blaZ-I-R*, *aacA-aphD*, *dfrA*, *tet(M)*, *sdrM*, *fosB*	*sea*, *seb*, *sek*, *seq*, *sak*, *scn*
787	EP	*blaZ-I-R*, *erm(C)*, *aacA-aphD*, *dfrA*, *tet(M)*, *sdrM*, *fosB*	*sea*, *seb*, *sek*, *seq*, *sak*, *scn*
823	EP	*blaZ-I-R*, *erm(C)*, *aacA-aphD*, *aadD*, *dfrA*, *tet(M)*, *sdrM*, *fosB*	*sea*, *seb*, *sek*, *seq*, *sak*, *scn*
58	EP	*blaZ-I-R*, *aacA-aphD*, *dfrA*, *tet(M)*, *sdrM*, *fosB*	*sea*, *seb*, *sek*, *seq*, *sak*, *scn*
55	IND	*blaZ-I-R*, *erm(C)*, *aacA-aphD*, *aadD*, *dfrA*, *tet(M)*, *sdrM*, *fosB*	*sea*, *seb*, *sek*, *seq*, *sak*, *scn*
389	MP	*blaZ-I-R*, *erm(C)*, *aacA-aphD*, *dfrA*, *tet(M)*, *sdrM*, *fosB*	*seb*, *sek*, *seq*
747	MPE	*blaZ-I-R*, *erm(C)*, *aacA-aphD*, *dfrA*, *tet(M)*, *sdrM*, *fosB*	*sea*, *seb*, *sek*, *seq*, *sak*, *scn*
612	CAP	*blaZ-I-R*, *aacA-aphD*, *tet(K)*, *sdrM*, *fosB*	*sea*, *seb*, *sek*, *seq*, *sak*, *scn*
734	EP	*blaZ-I-R*, *erm(C)*, *aacA-aphD*, *dfrA*, *tet(M)*, *sdrM*, *fosB*	*seb*, *sek*, *seq*, *sak*, *scn*
742	EP	*blaZ-I-R*, *erm(C)*, *aacA-aphD*, *dfrA*, *tet(M)*, *sdrM*, *fosB*	*sea*, *seb*, *sek*, *seq*, *sak*, *scn*
822	EP	*blaZ-I-R*, *aacA-aphD*, *dfrA*, *tet(M)*, *sdrM*, *fosB*	*sea*, *seb*, *sek*, *seq*, *sak*, *scn*
723	EP	*blaZ-I-R*, *erm(C)*, *aacA-aphD*, *dfrA*, *tet(M)*, *sdrM*, *fosB*	*sea*, *seb*, *sek*, *seq*, *sak*, *scn*
778	EP	*blaZ-I-R*, *erm(C)*, *aacA-aphD*, *dfrA*, *tet(M)*, *sdrM*, *fosB*	*sea*, *seb*, *sek*, *seq*, *sak*, *scn*
570	MP	*blaZ-I-R*, *aacA-aphD*, *dfrA*, *tet(M)*, *sdrM*, *fosB*	*sea*, *seb*, *sek*, *seq*, *sak*, *scn*
413	MPE	*blaZ-I-R*, *erm(C)*, *aacA-aphD*, *dfrA*, *tet(M)*, *sdrM*, *fosB*	*sea*, *seb*, *sek*, *seq*, *sak*, *scn*
716	MPE	*blaZ-I-R*, *aacA-aphD*, *dfrA*, *tet(M)*, *sdrM*, *fosB*	*sea*, *seb*, *sek*, *seq*, *sak*, *scn*
**Clonal Complex 22**			
34	CAP	*blaZ-I-R*, *vat(B)*, *fosB*	*seg*, *sei*, *sem*, *sen*, *seo*, *seq*, *seu*, *sak*, *chp*, *scn*
38	CAP	*blaZ-I-R*, *vat(B)*, *fosB*	*sec*, *seg*, *sei*, *sel*, *sem*, *sen*, *seo*, *seq*, *seu*, *sak*, *chp*, *scn*
253	CAP	*blaZ-I-R*, *fosB*	*seg*, *sei*, *sem*, *sen*, *seo*, *seq*, *seu*, *sak*, *chp*, *scn*
257	CAP	*blaZ-I-R*, *erm(C)*	*sec*, *seg*, *sei*, *sel*, *sem*, *sen*, *seo*, *seq*, *seu*
330	CAP	*blaZ-I-R*	*seg*, *sei*, *sem*, *sen*, *seo*, *seq*, *seu*
428	CAP	*blaZ-I-R*	*seg*, *sei*, *sem*, *sen*, *seo*, *seq*, *seu*, *chp*
606	CAP	*blaZ-I-R*	*sec*, *seg*, *sei*, *sel*, *sem*, *sen*, *seo*, *seq*, *seu*, *sak*, *chp*, *scn*
610	CAP	*blaZ-I-R*	*sec*, *seg*, *sei*, *sel*, *sem*, *seln*, *seo*, *seq*, *seu*
622	CAP	*blaZ-I-R*	*seg*, *sei*, *sem*, *sen*, *seo*, *seq*, *seu*, *sak*, *chp*, *scn*
844	CAP	*blaZ-I-R*	*seg*, *sei*, *sem*, *sen*, *seo*, *seq*, *seu*, *sak*, *chp*, *scn*
809	EP	*blaZ-I-R*	*sec*, *seg*, *sei*, *sel*, *sem*, *sen*, *seo*, *seq*, *seu*, *sak*, *chp*, *scn*
100	MPE	*blaZ-I-R*, *fosB*	*seg*, *sei*, *sem*, *sen*, *seo*, *seq*, *seu*, *sak*, *chp*, *scn*
**Clonal Complex 59**			
773	MPE	*blaZ-I-R*, *msr(A)*, *mph(C)*, *sdrM*	*sea*, *seb*, *sek*, *seq*, *sak*, *chp*, *scn*
**Clonal Complex 88**			
728	EP	*blaZ-I-R*, *erm(A)*, *sdrM*	*sec*, *sel*, *sak*, *scn*
815	EP	*blaZ-I-R*, *erm(A)*, *sdrM*	*sak*, *scn*
786	HC	*blaZ-I-R*, *erm(A)*, *sdrM*	*sec*, *sel*, *sak*, *scn*
103	MPE	*blaZ-I-R*, *sdrM*	*sec*, *sel*, *sak*, *scn*
**Clonal Complex 398**			
516	PP	*blaZ-I-R*, *erm(C)*, *tet(K)*, *tet(M)*, *sdrM*, *fosB*	*seg*

EP, equine practice; CAP, companion animal practice; MPE, mixed animal practice with both companion animals and equines; MP, mixed animal practice with companion animals and ruminants but no equines; PP, pig practice; HC, herd consulting practice; IND, industry veterinarian (pharmaceutical representative) with no clinical practice; STU, veterinary science student with undefined exposure.

### Virulence gene data

The carriage of virulence genes was also strongly influenced by the clonal complex (Table 2 and S1 File). The respective virulence gene profiles varied little for isolates belonging to CC8 and ST22. The majority of CC8 isolates possessed enterotoxin genes *seb+sek+seq* (24/24) (carried by *S*. *aureus* pathogenicity island 3 [SaPI3]) and the type D immune evasion cluster (IEC) (*sea*+*sak*+*scn*) (21/24). The 12 ST22 isolates possessed the enterotoxin *egc* cluster (*seg+sei+sem+sen+seo+seu)* carried by the ʊSaβ genomic island. Five of the isolates also harboured enterotoxins *sec+sel* (carried by SaPImw2). Eight isolates harboured a type B IEC (*sak+chp+scn*). None of the 44 isolates carried the toxic shock syndrome toxin 1 gene (*tst1*) or the Panton-Valentine Leukocidin (PVL) determinants *lukF* and *lukS*.

### Statistical analysis of associations between isolate source and clonal complex of isolates

After excluding from consideration those veterinarians having exposure to more than one class of animal (i.e. mixed animal practitioners), a very strong positive association was found between veterinarians working exclusively with companion animals and carriage of ST22 MRSA, and those working exclusively with horses and carriage of CC8 MRSA. Isolates belonging to ST22 were more than 50 times more likely to be carried by a companion animal practitioner than another type of veterinarian (OR = 52.5, 95% CI = 5.2–532.7, *P* < 0.001). Moreover, those belonging to CC8 were more than 17 times more likely to be carried by a veterinarian working exclusively with horses than another type of veterinarian (OR = 17.5, 95% CI = 3.3–92.5, *P* < 0.001).

## Discussion

Recent advances in molecular analyses have facilitated a quantum leap in our understanding of the epidemiology of MRSA in animals and humans intimately exposed to animals. This study considered the molecular and antimicrobial resistance characteristics of MRSA isolated from Australian veterinarians. Foremost of the outcomes from the analyses was the dominance of MRSA strains belonging to CC8 and ST22 and their associations with distinct practice types (i.e. equine and companion animals, respectively), and a paucity of ST398, with only a single isolate obtained from a specialist pig practice veterinarian. Additionally, CC8 isolates from veterinarians with exposure to equine practice were more likely to be resistant to gentamicin and rifampicin, whereas ST22 isolates from veterinarians with exposure to companion animal practice were more likely to be resistant to fluoroquinolones.

CC8 MRSA has been observed as a cause of community-associated clinical infection in humans in the United States [25]. This clonal lineage has been isolated from horses and equine veterinarians in North America [7] and Europe [26] and appears much more adapted for survival among equine populations than other animal-associated MRSA clones [27]. In Australia, multiple CC8 community associated MRSA (CA-MRSA) clones have been described, including ST8-IV [2B] and ST612-IV [2B], however, the isolates are typically only resistant to the beta-lactam antimicrobials [28].

ST22 is considered an epidemic MRSA strain among human populations in Europe, and has been isolated from companion animals and companion animal veterinarians in this region [1,29]. Similarly, ST22 MRSA is a leading cause of healthcare-associated MRSA infections in people in Australia [30] and has been isolated from Australian canines with skin and soft tissue infections (M. Barton, unpublished data). This clonal lineage is competent at “spilling over” from the human population into companion animals where it can persist for short periods of time, opportunistically cause clinical infections, and move bi-directionally [1].

The low prevalence of ST398 MRSA in veterinarians in this study is noteworthy as ST398 has been shown to be disseminated across multiple continents and colonises food animals, horses and people that are routinely exposed to animals [2,31]. ST398 MRSA has previously been observed among livestock veterinarians in Europe and North America, [5,32] with prevalence rates as high as 45% [5]. There are relatively few dedicated intensive livestock veterinarians in Australia in comparison to the main regions of intensive livestock production in Europe and North America. For example there are only about 100 members of the pig specialist interest group of the Australian Veterinary Association [33]. Annual surveillance studies of community-associated and healthcare-acquired MRSA infection have shown that ST398 MRSA is responsible for very few clinical cases of infection in humans in Australia [30,34]. However, ST398 MRSA was detected among pigs in Australia during a contemporaneous study [16]. It is reasonable to expect that the organism may have further disseminated among the Australia pig herd and other potential host species, including humans in subsequent years. A follow up survey therefore seems warranted to determine if the prevalence has increased among veterinarians.

The high MRSA colonisation rates in companion animal and equine practice veterinarians in Australia could be associated with the handling and administration of antimicrobials in these animal species. This notion is supported by the high proportion of ST22 isolates showing resistance to fluoroquinolones, which is almost exclusively used in companion animals in Australia [35]. Moreover, a high proportion of CC8 isolates were resistant to gentamicin, which is frequently administered empirically to horses in Australia, and rifampicin which is exclusively used in combination with a macrolide to treat *Rhodococcus equi* infections in foals [14,36]. Use of both antibiotics in horses (gentamicin by intravenous injection and rifampicin by oral administration to foals) could result in considerable opportunity for the veterinarian to be exposed to low levels of antimicrobials which could potentially influence the high prevalence of MRSA carriage in the Australian equine veterinary community [14,37].

In addition to the CC8, ST22 and ST398 isolates a small number of CA-MRSA clones were identified including ST1-IV [2B] (WA1), in a veterinary student with undefined exposure, as well as two ST78-IV [2B] (WA2) isolates and one ST59-IV [2B]&5 (WA15). These three clones, particularly WA1 and WA2, are frequently isolated in Australia and therefore their detection is probably not associated with working in the animal industry [38,39]. The USA100 isolate (ST5-II [2A]), is a major healthcare-associated clone in North America and in Japan/Korea. Rarely identified in Australia, patients colonised or infected with USA100 are usually epidemiologically linked to North America [40].

### Conclusions

This study informed on the molecular and antimicrobial resistance characteristics of MRSA in veterinarians in Australia, allowing isolates to be placed into epidemiological and temporal context with strains from other sources both in Australia and abroad. Veterinarians predominantly carried MRSA CC8 and ST22, archetypes that have over the past decade become prolific colonisers, and under certain circumstances causes of clinical infections, in humans and some domestic species, worldwide. These findings may have implications for biosecurity and infection control programs in veterinary and public health settings in Australia.

## Supporting Information

S1 File The S1 File contains the full data set including isolate origin, clonal association, resistance and virulence gene data for all the isolates described in this study. (XLSX)
